# Recent progress of heterocycle ring‐opening (co)polymerization for the synthesis of sequence‐controlled block polyesters and polycarbonates

**DOI:** 10.1002/smo.20240057

**Published:** 2024-12-01

**Authors:** Hongyu Zhao, Chenyang Hu, Xuan Pang, Xuesi Chen

**Affiliations:** ^1^ Key Laboratory of Polymer Ecomaterials Changchun Institute of Applied Chemistry Chinese Academy of Sciences Changchun China; ^2^ School of Applied Chemistry and Engineering University of Science and Technology of China Hefei China

**Keywords:** aliphatic polycarbonates, aliphatic polyesters, block copolymers, heterocycle monomers, ring‐opening (co)polymerization

## Abstract

Aliphatic polyesters and polycarbonates are among the promising sustainable polymers, which exhibit unique degradability and chain‐chain interactions owing to their heterofunctionality. However, monocomponent aliphatic polyesters and polycarbonates usually suffer from inferior properties and functionalities. By contrast, precisely modulated block copolymers composed of polyesters and polycarbonates give rise to sustainable materials with tailored performance. An efficient approach to synthesize the block copolymers is the ring‐opening (co)polymerization of the heterocycle monomers. Herein, this review presents the heterocycle monomer ring‐opening (co)polymerization for the formation of sequence‐controlled block polyesters and polycarbonates. Available synthetic strategies, different monomers, monomer combinations and the catalyst systems for the formation of different block polyesters and polycarbonates are summarized.

## Graphical abstract

Block copolymers are fundamental for the development of smart molecules. Aliphatic polyesters and polycarbonates have received attention as heteroatom‐containing polymers that are biodegradable and biocompatible. Block polymers from polyesters and polycarbonates have the ability to form smart molecules. This review presents the heterocycle monomer ring‐opening (co)polymerization for the synthesis of sequence‐controlled block polyesters and polycarbonates.

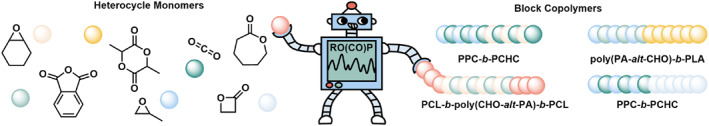

## 1 INTRODUCTION

Polymers play a dominant role in modern life from commodity products to high‐tech applications. Among a variety of polymers, block copolymers are fundamental for the development of smart molecules with tailored‐made functionalities. Aliphatic polyesters and polycarbonates have received significant attention as heteroatom‐containing polymers with biodegradability and biocompatibility.^[1]^ Poly(lactic acid) (PLA) is a representative commercial aliphatic polyester, which is produced from the oxygen‐heterocyclic monomer lactide (LA) ring‐opening polymerization (ROP). Currently, PLA has been utilized for packaging and fibers. Aliphatic polycarbonates are usually made through the ring‐opening copolymerization (ROCOP) of epoxide with carbon dioxide (CO_2_). They have also attracted considerable attention in recent years because these polymers exhibit lower consumption of petrochemical crude materials while decreasing CO_2_ emissions in all life cycle.^[2]^

**FIGURE 1:**
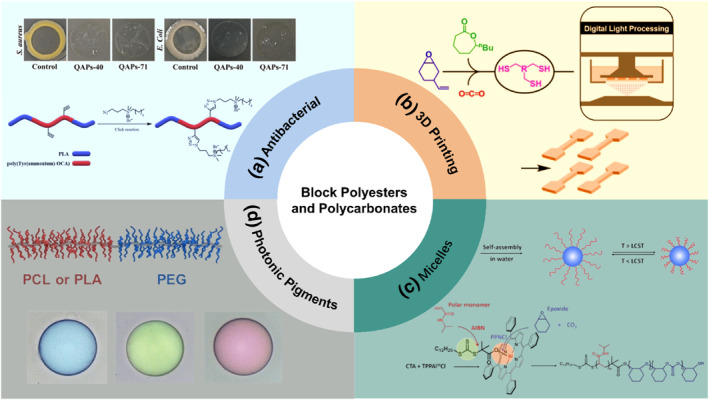
The applications of the resulting block polyesters and polycarbonates. (a) Antibacterial. Reproduced with permission.^[7]^ Copyright 2021, John Wiley and Sons. (b) 3D Printing. Reproduced under terms of the CC‐BY license.^[8]^ Copyright 2024, John Wiley and Sons. (c) Micelles. Reproduced with permission.^[9]^ Copyright 2023, John Wiley and Sons. (d) Photonic pigments. Reproduced with permission.^[10]^ Copyright 2022, John Wiley and Sons.

However, compared to polymers that contain aromatic/rigid functionalities in the polymer backbone, aliphatic polyesters and polycarbonates exhibit modest physical and chemical properties.^[3]^ One approach to tuning polyesters and polycarbonates properties is by incorporating the other blocks into the polymer chain to alter polymer backbone functionality. Moreover, the block polyesters and polycarbonates usually have different performance, in contrast to the blend of the constituent homopolymers. For example, the block copolymers of PLA and poly(propylene carbonate) (PPC) exhibit superior thermal and mechanical properties, which beneficially combine the high glass transformation temperature (*T*_g_) and good extension strength of PLA with the tensile toughness of PPC.[^4^, ^5^, ^6^] Moreover, the sequence‐controlled block polyesters and polycarbonates have some potential applications, such as antibacterial, additive manufacturing, micelles, or photonic pigments (Figure 1).[^7^, ^8^, ^9^, ^10^]

Heteroatom‐containing polymers are currently undergoing a rise due to they have the potential to monomer–polymer equilibria such as complete depolymerization, chemical recycling, and even biodegradation.[^11^, ^12^] Polyesters and polycarbonates are the typical heteroatom‐containing polymers. The ROCOP of epoxides/anhydrides as the typical heterocycle ROCOP to obtain polyester has been extensively researched from last century.[^13^, ^14^] The incorporation of two distinct kinds of heterocycle monomers significantly greatly increases the variety of the possible polymer structures, resulting in over 400 possible polymer structures.^[1]^ Furthermore, the heterocycle monomer containing N, S atoms ring‐opening (co)polymerization has also been studied in the recent years. The polymers containing N, S heteroatoms show special properties, which have the potential to be expanded into various sectors including ionic conductors, optical materials and medical materials.^[12]^

Depending on the advancement of polymerization craftsmanship, polyesters and polycarbonates could be synthesized from chain growth polymerization and polycondensation.^[15]^ Compared with condensation polymerization, chain growth polymerization provides an effective way to produce polymers without the removal or addition of other small molecules. As the appealing approaches of chain growth polymerization, ROP and ROCOP offer the controlled polymerization routes to obtain aliphatic polyesters and polycarbonates with precise polymer molecular weights, narrow dispersities, architectures, compositions, and well‐defined end‐groups.

**SCHEME 1:**
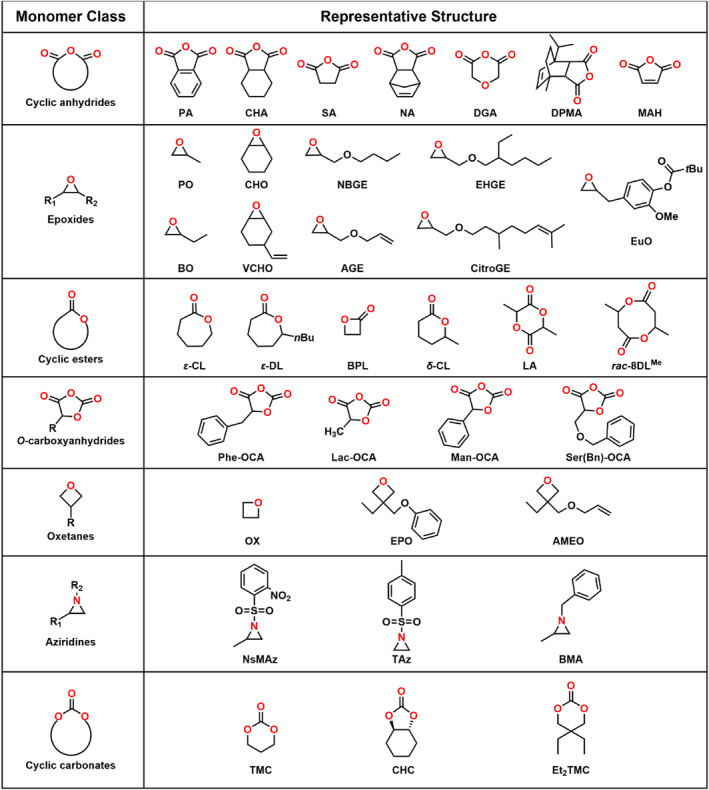
The heterocycle monomers used for the synthesis of polyester and polycarbonate blocks. AGE, allyl glycidyl ether; AMEO, 3‐((allyloxy)methyl)‐3‐ethyloxetane; BMA, 1‐benzyl‐2‐methylaziridine; BO, 1,2‐butylene oxide; BPL, *β*‐propiolactone; CHA, 1,2‐cyclohexanedicarboxylic anhydride; CHC, cyclohexe carbonate; CHO, cyclohexene oxide; CitroGE, citronellyl glycidyl ether; DGA, diglycolide anhydride; DPMA, *rac‐cis‐endo‐*1‐isopropyl‐4‐methyl bicyclo[2.2.2]oct‐5‐ene‐2,3‐dicarboxylic anhydride; EHGE, 2‐ethylhexyl glycidyl ether; EPO, 3‐ethyl‐3‐(phenoxymethyl)oxetane; Et_2_TMC, 2,2‐diethyltrimethylene carbonate; EuO, eugenol epoxide; LA, lactide; MAH, maleic anhydride; NA, norbornene anhydride; NBGE, *n*‐butyl glycidyl ether; NsMAz, 1‐(2‐nitrobenzenesulfonyl) 2‐methyl‐aziridine; OCA, *O*‐carboxyanhydride; OX, oxetane; PA, phthalic anhydride; PO, propylene oxide; *rac*‐8DL^Me^, *rac*‐eight‐membered cyclic diolides (Me = methyl); SA, succinic anhydride; TAz, *N*‐tosylaziridine; TMC, trimethylene carbonate; VCHO, vinyl cyclohexene oxide; *δ*‐CL, *δ*‐caprolactone; *ε*‐CL, *ε*‐caprolactone; *ε*‐DL, *ε*‐decalactone.

**SCHEME 2:**
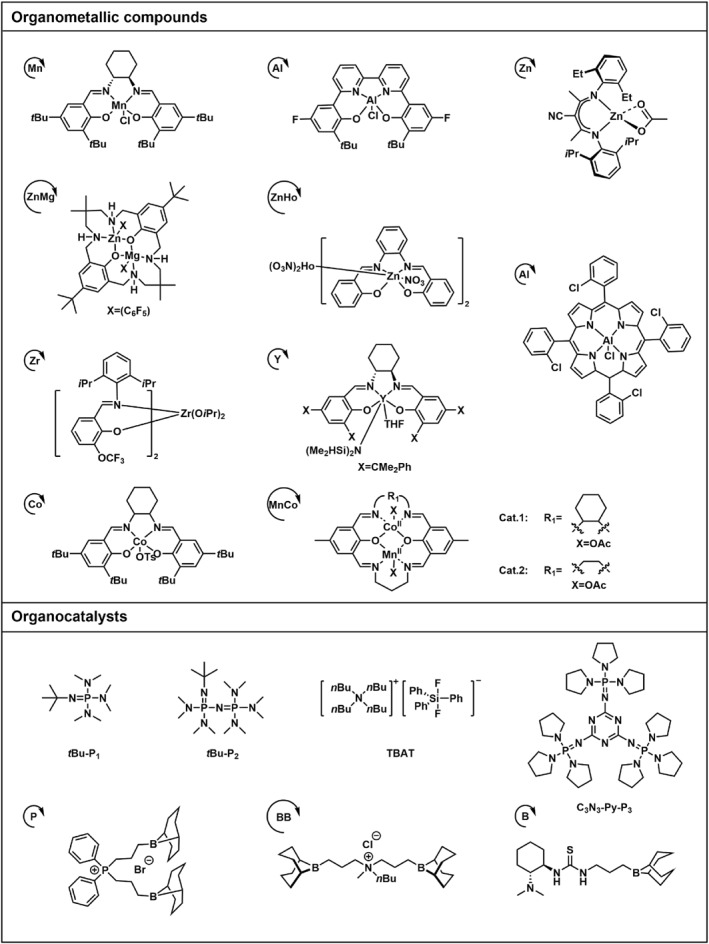
The representative catalysts for the heterocycle ring‐opening (co)polymerization.

This review presents the formation of sequence‐controlled block polyesters and polycarbonates through the heterocycle monomer ring‐opening (co)polymerization. It focuses on the available strategies for the formation of varying block copolymers via kinetic control over the monomer mixture, sequential addition of monomers or controlled switchable polymerization by external stimuli. Otherwise, the different monomers, monomer combinations and the catalyst systems covering organometallic compounds and organocatalysts are also discussed in this review. The performance of various catalysts in catalysis of polyesters and polycarbonates formation and the application of the block polyesters and polycarbonates have been reviewed elsewhere and are not discussed in this review.[^16^, ^17^] Representative structure of the heterocycle monomers and representative catalysts, which were presented in this review, are shown in Schemes 1 and 2.

## 2 ROCOPs INVOLVING ANHYDRIDE AND EPOXIDE

The copolymerization of anhydride and epoxide allows for the controlled synthesis of the polyesters. The polyesters with precise polymer molar mass and narrow dispersity offer a controlled platform to synthesize block copolymer.[^1^, ^3^] To obtain functionalized block polyester with diverse structure, it is important to introduce a third monomer different from anhydride and epoxide in the polymerization.

### 2.1 Anhydride/epoxide/cyclic ester

**SCHEME 3:**
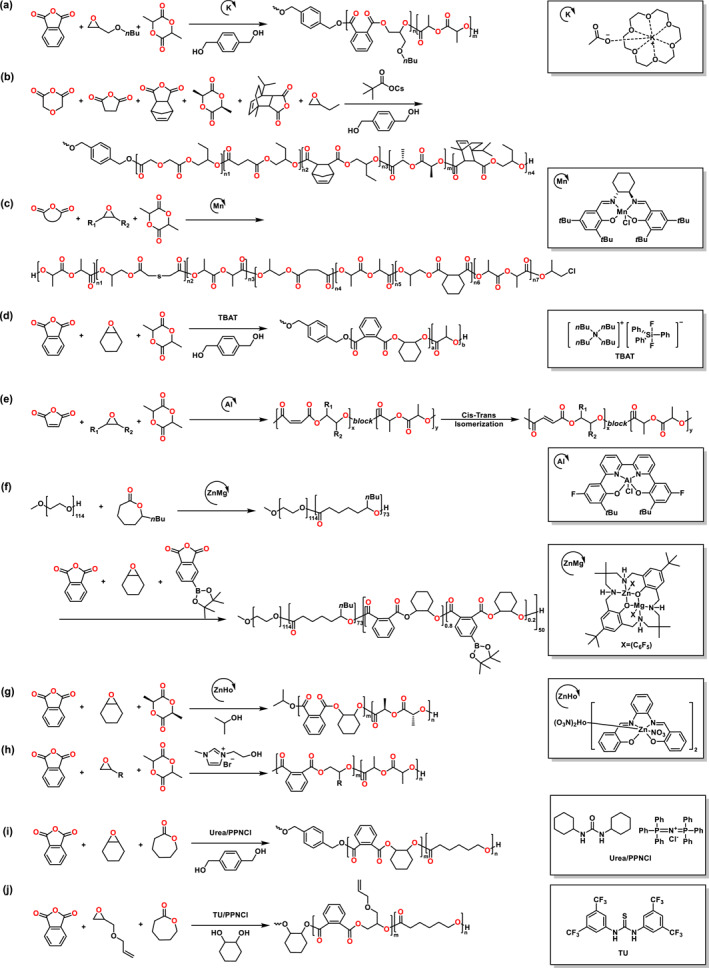
Ring‐opening copolymerization of (a–e, g, h) anhydride, epoxide, lactide and (f, i, j) anhydride, epoxide, lactone.

Alkali metal carboxylates have been utilized for the ROP of cyclic esters as catalysts.[^18^, ^19^] The Li group reported that alkali metal carboxylates worked as the initiators in the ROCOP of anhydrides and epoxides.^[20]^ Subsequently, Li and coworkers combined the 18‐Crown‐6‐ether with potassium acetate to obtain diverse polyester polyols with high end‐group fidelity (Scheme 3a).^[21]^ These polydiols could be efficiently prepared at an extremely high monomer feed ratio with only 0.0004 mol% catalyst loading. Moreover, the block polyols could be synthesized in one‐pot among anhydrides, epoxides, and cyclic esters. The block polyols had controllable molecular weights, narrow dispersities and monomodal molar mass distributions.

In 2021, the Satoh group developed a self‐switchable polymerization pathway to prepare the sequentially and architecturally controlled multiblock polyesters by alkali metal carboxylate catalysts.^[22]^ Depending on the reactivity trend of ROCOP of epoxides and anhydrides and ROP of LA, various types of complex block copolymers could be simply prepared. Subsequently, they demonstrated a system for synthesizing multiblock copolymers involving up to 11 blocks (Scheme 3b).^[23]^ The catalyst linked five different catalytic cycles, comprising four ROCOPs of anhydrides/epoxides and the ROP of cyclic ester, in the six‐component system. The block copolymers with 11 blocks had the *M*_n_s as many as 7.8 kg mol^−1^ and *Đ* ∼ 1.46. Trimellitic anhydride, a functional anhydride, was used to prepare a multiblock copolymer with a core–shell structure in the one step.

In 2023, the Chen group reported the synthesis of multiblock copolymers by multi‐switching copolymerization consisting of anhydrides, epoxides, and LA (Scheme 3c).^[24]^ The copolymers were obtained with a maximum of seven blocks by addition of anhydrides sequentially, without the chain transfer agent (CTA) or cocatalyst. In addition, the gel permeation chromatography (GPC) traces demonstrated increasing *M*_n_s from 26.2 to 45.8 kg mol^−1^ with narrow dispersities as the process of the reaction. Very recently, the Chen group reported multi‐task organofluoride catalysts for the preparation of polyesters, which could be accomplished either via ROP of LA or ROCOP between anhydrides and epoxides (Scheme 3d).^[25]^ Furthermore, terpolymerization of anhydrides, epoxides and LA could be constructed through an organofluoride catalyst to yield a block copolymer with a strictly controlled polymerization sequence. In addition, the organofluoride catalysts had high reactivity for the depolymerization of PLA.

Li and coworkers introduced a well‐defined unsaturated polyester block into the copolymer through an Al complex switchable copolymerization involving maleic anhydride, LA and epoxide mixtures (Scheme 3e).^[26]^ For the unsaturated polyester block, the cis–alkene groups could completely convert to trans‐alkene groups through isomerization. In 2023, the other group demonstrated that the ROCOP of propylene oxide (PO), maleic anhydride and glycolid depended on a Cr complex.^[27]^ The obtained poly(propylene maleate)‐*block*‐polyglycolide had a *M*_n_ of 3.5 kg mol^−1^ and *Đ* ∼ 1.33.

The heterodinuclear Zn/Mg complex was reported to prepare ABC block copolymer by Williams group (Scheme 3f).^[28]^ The polymerization started with the ROP of *ε*‐decalactone (*ε*‐DL) in a mixture involving cyclohexene oxide (CHO) and toluene, which was initiated using mPEG_114_−OH. With the addition of a blend of BPin‐phthalic anhydride and phthalic anhydride (PA) in a single step, the obtained ABC block copolymer demonstrated an increase in molecular weight maintaining narrow dispersity and monomodal molar mass distribution (*M*_n_ of 20.4 kg mol^−1^ and *Đ* of 1.19). Moreover, the block copolymers with the amphiphile structures could be utilized to incorporate fluorescent markers through cross‐coupling processes. In 2022, the Chen group used a heterometallic Zn/Ho complex to synthesize well‐defined block copolymers among monomer mixtures of anhydride, epoxide and _L_‐LA (Scheme 3g).^[29]^ *M*_n_s of samples increased linearly with narrow dispersities with the extension of reaction time.

Ionic liquids (ILs), which contain different structures of anions and large organic cations, are commonly defined as liquid electrolytes due to the poorly coordinated ions.^[30]^ As a metal‐free catalyst, ILs have continued to attract huge interest in the ROP and ring‐opening alternating copolymerization (ROAC). The Song group used the hydroxyl functionalized IL to prepare sequence‐controlled copolyesters through polymerization among epoxides, PA and LA in the one‐pot (Scheme 3h).^[31]^ Organocatalysts and ILs could produce block polyesters from a mixture of CHO, PA, and _L_‐LA, but the organometallic Zn/Ho complex exhibited higher catalytic activity. However, the ILs were effective in switching the polymerization process between the ROCOP of CHO/PA and the ROP of _L_‐LA. Recently, they reported the other imidazolium IL catalysts to develop a copolymerization system of different anhydrides, epoxides and lactones.^[32]^ The copolymer of PA, PO, caprolactone had *M*_n_ of 21.8 kg mol^−1^ with *Đ* of 1.18.

The Meng group used dual urea and organic base binary catalytic system to construct multiblock copolymers from epoxides, PA and *ε*‐caprolactone (*ε*‐CL) through one‐step switchable polymerization (Scheme 3i).^[33]^ Triblock copolymers were obtained from a switchable terpolymerization of the ROCOP of epoxide with anhydride and the ROP of lactones. In 2023, the Zhu group detailed a thiourea (TU)/bis(triphenylphosphine)iminium chloride (PPNCl) binary catalytic system that could produce the well‐controlled block polyesters with a various series of structures from epoxides, cyclic anhydrides, and cyclic ester (Scheme 3j).^[34]^ Thermogravimetric analysis showed that the obtained block copolymers had degradation temperatures over 280°C with similar decomposition behavior.

### 2.2 Anhydride/epoxide/cyclic carbonate

**SCHEME 4:**
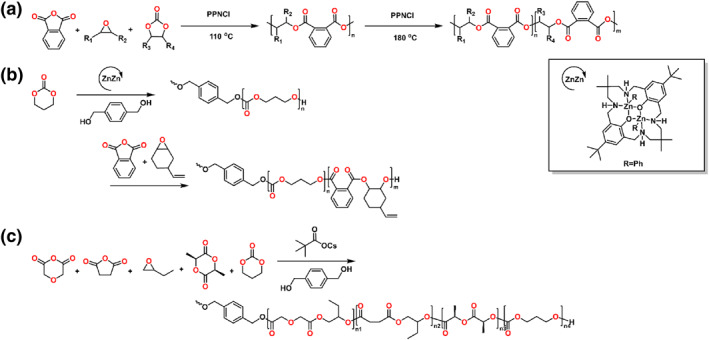
Ring‐opening copolymerization of (a) anhydride, epoxide, five‐membered cyclic carbonates and (b, c) anhydride, epoxide, trimethylene carbonate.

It is hard to polymerize five‐membered cyclic carbonates by ring‐opening (co)polymerization because the five‐membered rings exhibit low strain. The Wang group utilized the deprotection chemistry of CO_2_ to develop a facile temperature‐programmed strategy to construct precise block copolymers by organocatalyst (Scheme 4a).^[35]^ For the representative system consisting of CHO/propylene carbonate (PC)/PA, the poly(CHO‐*alt*‐PA) was first obtained up to 110°C via ROCOP of CHO/PA. When the temperature up to the 180°C, PO was released from PC and copolymerized with PA in order to obtain poly(PO‐alt‐PA) block. With the subsequent polymerization of PC/PA, the molecular weight region of GPC curves shifted to higher values with the similar molecular weight distribution.

The Williams group reported the block poly(carbonate‐ester) ionomers with impressive elasticity (>800%), tensile strengths (60 MPa) and recovery (95%) (Scheme 4b).^[36]^ Poly(trimethylene carbonate) (PTMC) was obtained through the ROP of trimethylene carbonate (TMC). PA/vinyl cyclohexene oxide (VCHO) were added into the reaction mixture to form the end blocks of the triblock copolymer, which was referred to as PE(v). There was only PE(v) end group present without any signal of PTMC from the analysis of the end group of PE(v)‐*b*‐PTMC‐*b*‐PE(v), a typical triblock copolymer in this work. Moreover, the GPC traces demonstrated a consistent increase in *M*_n_ throughout the reaction with narrow dispersity and unimodal molar mass distribution. Satoh and coworkers obtained the heptablock copolymers through a simple alkyl metal carboxylate catalyst among anhydride/epoxide/cyclic carbonate/LA via one‐step polymerization (Scheme 4c).^[37]^ Diffusion‐ordered spectroscopy (DOSY) NMR manifested one signal diffusion coefficient, which confirmed the polymer was a copolymer not a blend.

### 2.3 Anhydride/epoxide/oxetane

**SCHEME 5:**
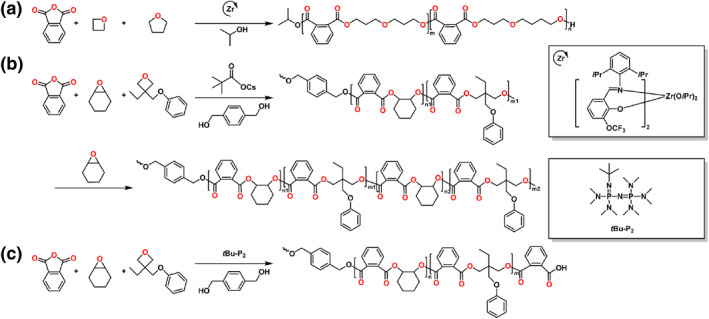
Ring‐opening copolymerization of (a) anhydride, oxetane, tetrahydrofuran and (b, c) anhydride, epoxide, 3‐ethyl‐3‐(phenoxymethyl)oxetane.

Oxetane is a four‐membered heterocyclic molecule, which can be produced through 1,3‐propylene glycol.^[38]^ As strained cyclic ethers, the oxetane has received enormous interest for the polymerization of polyester and polycarbonate. The ROCOP of anhydride and oxetanes is more challenging than the ROCOP of anhydride and epoxide because of the ring tension of the oxetanes. Buchard and coworkers demonstrated the ROCOP of cyclic anhydrides with an oxetane comonomer through salen catalysts.^[39]^ Block copolymers with the well‐defined blocks were synthesized by sequential addition of different anhydrides. In 2022, Williams and coworkers demonstrated a Zr complex that catalyzes the ROCOP of anhydrides (A) with epoxides/oxetane (B) to make the ABB‐poly(ester‐*alt*‐ethers) with ∼1:1 integrals of the methylene‐ester (−COOCH_2_CH−) and methylene‐ether (−OCH_2_CH−) (Scheme 5a).^[40]^

The Satoh group used alkali metal carboxylate as a simple catalyst to synthesize the well‐defined multiblock copolymers from a blend of anhydrides, epoxides and oxetane, connecting the ROAC of anhydride/oxetane and anhydrides/epoxides (Scheme 5b).^[41]^ Based on the great difference in reactivity between oxetane and epoxide and the reactivity gradient of different anhydrides, diverse block copolymers were synthesized using the one‐step procedure. Pentablock tetrapolymers with <3% tapered section were generated by tuning the stoichiometric ratio of the monomers. Recently, the Satoh group reported the organobase‐catalyzed ROCOP of anhydride and oxetane, which can be integrated with the ROAC of PA/epoxide and the ROP of valerolactone (VL), TMC, or _L_‐lactide (Scheme 5c).^[42]^ A P(PA‐*alt*‐EPO)‐*b*‐P(PA‐*alt*‐CHO)‐*b*‐P(PA‐*alt*‐EPO) triblock copolymer with a narrow tapered region was synthesized by *t*Bu‐P_2_ under 130°C, which exhibited higher reactivity than the alkali metal carboxylate catalyst. Moreover, in the ROCOP of PA and trimethylene oxide, the turnover frequency obtained using the *t*Bu‐P_2_ was approximately seven times higher than that obtained using cesium pivalate.

### 2.4 Anhydride/epoxide/aziridine

**SCHEME 6:**
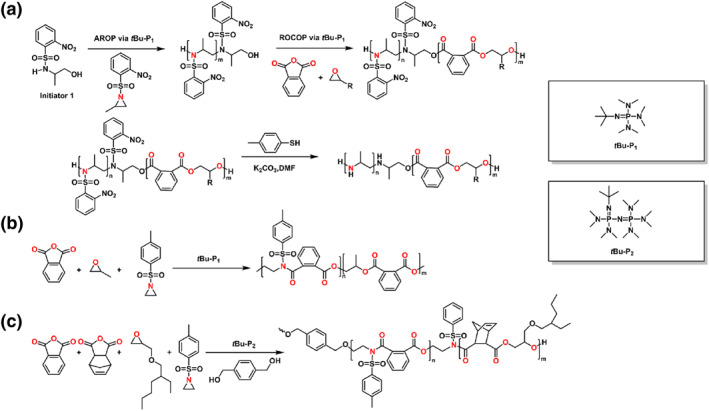
Ring‐opening copolymerization of (a) anhydride, epoxide, 1‐(2‐nitrobenzenesulfonyl) 2‐methyl‐aziridine and (b, c) anhydride, epoxide, N‐sulfonyl aziridine by phosphazene‐based catalysts.

Aziridine with the analogous structure as the oxirane, but the ROP of the aziridine is more challenging than that of oxirane. For the polymerization of aziridine, the electron‐withdrawing groups are introduced into the aziridine, which can be reductively removed to give the corresponding polyimine.^[43]^ A study detailed the copolymerization of *N*‐sulfonyl aziridine (*N*‐tosylaziridine, TAz) and PA catalyzed by halide salts.^[44]^ Chen and coworkers reported the telechelic block copolymer combined poly(propylene oxide‐*alt*‐phthalic anhydride) and linear polymers polypropylenimine using single phosphazene organobase catalyst (Scheme 6a).^[45]^ The GPC traces of the final polymers after desulfonylation clearly shifted to lower molecular weights and the dispersity was still low (*Đ* < 1.20). Compared to the original polymer, the telechelic copolymer demonstrated obvious hydrophilicity.

The Hadjichristidis group reported the highly selective organocatalytic polymerization of multiblock alternating copolymers among a monomer mixtures of PA, PO and TAz via one‐pot/one‐step (Scheme 6b).^[46]^ The Satoh group developed a one‐step polymerization system by *t*Bu‐P_2_ for the formation of varying block copolymers, which consisted of the poly(amide ester)s (Scheme 6c).^[47]^ The self‐switchable polymerization consisted of the ROAC of epoxide/anhydride and TAz/anhydride to synthesize poly(amide ester)‐*b*‐polyester. These block copolymers exhibited two *T*_g_ values, which were indicative of microphase separation as confirmed.

### 2.5 Anhydride/epoxide/vinyl monomer

**SCHEME 7:**
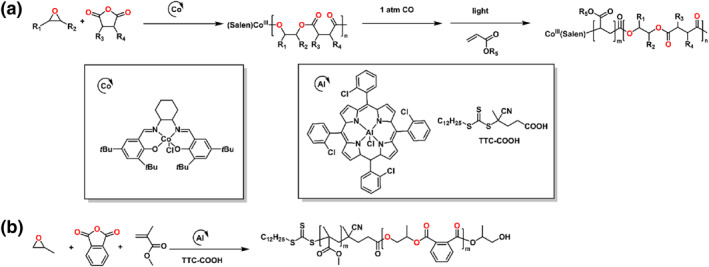
Ring‐opening copolymerization of (a) anhydride, epoxide, acrylate and (b) anhydride, epoxide, methyl methacrylate by organometallic compounds.

In 2020, the Zhou and Wang group reported a series of polyester‐*b*‐polyacrylate multiblock copolymers, which achieved through one‐pot copolymerization of the cyclic anhydrides/epoxides/acrylates (Scheme 7a).^[48]^ The carbon monoxide (CO) was introduced into the polymerization to switch the mechanism from ROCOP of anhydrides and epoxides to organometallic mediated controlled radical polymerization (OMRP) of acrylates. Further, the polymerization could be regulated by visible light, giving ON/OFF regulation of living chain growth in OMRP. Subsequently, this group demonstrated the Al complex catalyzed reversible addition‐fragmentation chain transfer polymerization (RAFT) of vinyl monomers and ROAC of anhydrides/epoxides in a sequential or simultaneous way (Scheme 7b).^[49]^ The diblock copolymers could be synthesized from monomer mixtures with predictable molecular weights and narrow distributions. As compared with the Co analogs, the Al complex could mediate auto‐tandem catalysis for the ROAC of anhydrides/epoxides and RAFT of vinyl monomers via photoinduced electron/energy transfer.

## 3 ROCOPs INVOLVING CO_2_ AND EPOXIDE

Inspired by the chemosimilarity between CO_2_ and anhydride, ROCOP of these two monomers with epoxides has always been discussed together. Many catalysts are capable of catalyzing the formation of both polyester and polycarbonate. Utilizing epoxides in combination with CO_2_ to obtain polycarbonates has attracted considerable attention in commodity and medical applications, due to their excellent biocompatibility and tunable degradation.^[3]^

### 3.1 CO_2_/epoxide

**SCHEME 8:**
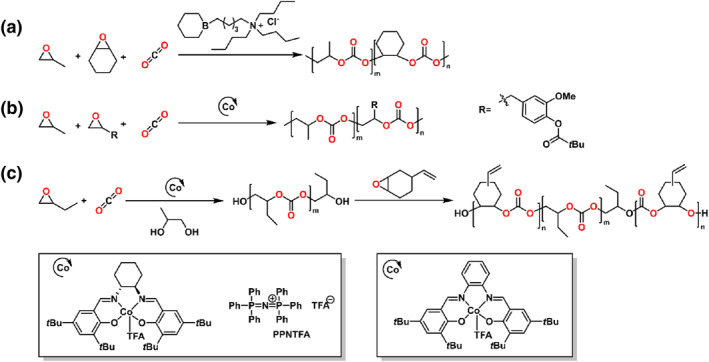
Ring‐opening copolymerization of CO_2_ and epoxide (a) by organocatalysts and (b, c) by organometallic compounds.

Gnanou and coworkers demonstrated a range of bifunctional organoboron catalysts, involving an ammonium salt, for effective ROAC of epoxides with CO_2_ in high polycarbonate productivities (Scheme 8a).^[50]^ Through the ROAC of PO and CHO with CO_2_, PPC and poly(cyclohexene carbonate) (PCHC) could be synthesized. By chain extension of PPC with CHO, a well‐controlled PPC‐*b*‐PCHC diblock copolymer could be obtained. This organoboron catalyst enabled the ROAC of epoxides and CO_2_ as impressive productivities; 1 g catalyst could yield 271.5 g of PPC and 5.7 kg of PCHC.

The Darensbourg and Bhat group demonstrated the formation of terpolymers generated from the copolymerization among eugenol epoxide, PO and CO_2_ by binary salen catalyst system (Scheme 8b).^[51]^ In their previous work, when eugenol epoxide coupled with CO_2_, only the cyclic carbonate could be afforded.^[52]^ However, with the addition of PO, the eugenol epoxide copolymerized with CO_2_ to provide terpolymers or diblock copolymers. Depending on the relative ratios of eugenol epoxide and PO, the resulting polycarbonate terpolymers had different compositions. The *T*_g_ of the terpolymer increased with the increasing of the eugenol epoxide proportion. The same group utilized the other binary salen catalyst system to produce a range of triblock copolymers involving hard and soft blocks with adjustable polymer thermal and mechanical properties (Scheme 8c).^[53]^ These ABA polymers were obtained from ROCOP of butylene oxide/CO_2_ and ROCP of VCHO/CO_2_ with narrow distribution of molar masses. Moreover, the polymers could be 3D printed and modified or crosslinked via a thiolene click reaction.

### 3.2 CO_2_/epoxide/anhydride

**SCHEME 9:**
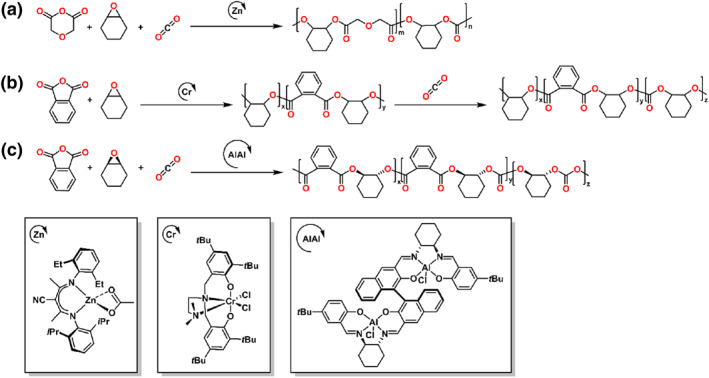
Ring‐opening copolymerization of CO_2_, epoxide, anhydride (a, c) in one pot and (b) via two steps by organometallic compounds.

In 2008, Coates and coworkers demonstrated the ROAC of CO_2_, epoxides and anhydrides by the Zn complex to produce the block poly(ester‐*b*‐carbonates) (Scheme 9a).^[54]^ Subsequently, a series of metal complexes and organocatalysts were studied in such terpolymerization.^[13]^

Recently, researchers also investigated novel catalytic systems in the terpolymerization of CO_2_, epoxides and anhydrides for the synthesis of diblock copolymers. The Cr complex with PPNCl was used for the copolymerization of poly(PA‐*alt*‐CHO)‐*b*‐PCHC (Scheme 9b).^[55]^ Capacchione and coworkers reported dinuclear Cr/Fe complexes for the formation of various epoxides with CO_2_ and their terpolymerization with PA.[^56^, ^57^]

In 2023, the Lu group reported that the enantiopure bimetallic Al complex mediated the asymmetric copolymerization of PA, CO_2_ and meso‐epoxides (Scheme 9c).^[58]^ The terpolymers exhibited unusual distributions of carbonate/ester units and excellent enantioselectivities that were unaffected by the carbonate‐ester distribution. The terpolymer exhibited a *T*_g_ of 89°C with 24% ester content and one melting point of about 237°C with a 68% ester content, which is similar to the isotactic CPO/PA copolymer (*T*_g_ = 89°C and melting point = 240°C).

**SCHEME 10:**
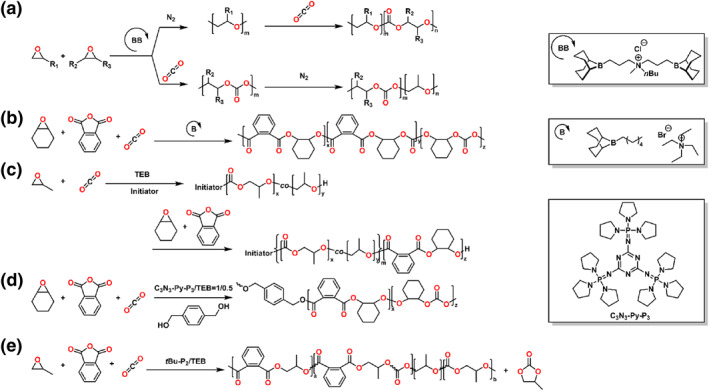
Ring‐opening copolymerization of CO_2_, epoxide, anhydride (a, c) via two steps and (b, d, e) in one pot by organocatalysts.

The Wu group reported a series of organoborane catalysts; these catalysts showed superior activities in polymerization of several epoxide‐involved transformations. In 2022, bifunctional quaternary ammonium‐containing boron‐based catalysts had been developed to yield well‐controlled polyether‐*block*‐polycarbonate copolymers under CO_2_ or N_2_ atmospheres from mixtures of epoxides (Scheme 10a).^[59]^ Very recently, they showed the investigation of the binary and bifunctional organoborane catalyst for terpolymerization of CHO/PA/CO_2_ (Scheme 10b).^[60]^ While tapering diblock copolymers were produced through the binary system, the bifunctional catalyst enabled the synthesis of the gradient polyester‐polycarbonate copolymers with different polyester content. The polymers had the *M*_n_s up to 7.8 kg mol^−1^ with narrow dispersity.

Very recently, the Feng and Gnanou group reported a range of polycarbonate‐based triblock copolymers by triethylborane (TEB)‐mediated polymerization (Scheme 10c).^[61]^ The resulting polymers could mechanically withstand high temperature with both linear and star‐shaped architectures. As soft blocks, a range of the varying contents of carbonate in poly(propylene oxide‐*co*‐propylene carbonate) (PPPC) were first prepared under low pressure of CO_2_, subsequently the CHO and PA were added to obtain triblock copolymers as hard poly(cyclohexene phthalate) (PCHPE) blocks. With the bis(triphenylphosphine)iminium sulfonylbisphenolate as the initiator, the well‐defined PCHPE and poly(ether carbonate) triblock copolymers had the *M*_n_s as high as 171 kg mol^−1^ and narrow dispersities.

Utilizing the PPNCl/TEB catalytic system could synthesize diblock and triblock poly(ester‐carbonate)s from epoxides, PA and CO_2_.^[62]^ Li and coworkers prepared diblock poly(ester‐carbonate) copolymers with a tapered structure through a similar catalytic system.^[63]^ In 2023, they demonstrated the chemoselective copolymerization to obtain polymer materials with diverse sequential structures and compositions through the varying stoichiometric ratio of phosphazene/TEB (Scheme 10d).^[64]^ With the stoichiometric ratio of C_3_N_3_‐Py‐P_3_/TEB = 1/0.5, a well‐controlled multiblock PCHC‐*b*‐poly(PA‐*alt*‐CHO)‐*b*‐PCHC copolymer with no gradient structure or tapered structure was obtained for the first time by ROCOP from a mixture of CHO, PA and CO_2_. These polymers generated from different reaction times showed steadily increasing *M*_n_ and narrow dispersity. Random or tapered poly(ester‐*co*‐carbonate) copolymers were otained by tuning the stoichiometric ratio of C_3_N_3_‐Py‐P_3_ to TEB. Moreover, the linear, three‐/four‐arm star terpolymers with well‐defined block structures and 45–61 mol% of PC could also be synthesized by different initiators. In addition, the binary organocatalytic system could synthesize block poly(carbonate‐ester) copolymers without tapered structures by one‐step/one‐pot methodology, which was previously only achieved through sequential addition of monomers by organometallic compounds and organocatalysts.

The Meng group reported the TEB‐activated ROCOP of ethylene oxide/CO_2_ targeted to obtain triblock copolymers with varying sequence structures containing poly(ethylene carbonate) (PEC) segments (Scheme 10e).^[65]^ A range of PEC‐PCHC‐PEC terpolymers with *M*_n_s up to 275 kg mol^−1^ had better thermal stabilities than PEC (*T*_5%_ = 158.0°C). The same group combined the different tertiary amine with TEB to produce diversified poly(ester‐*co*‐carbonate)s among CO_2_, anhydrides and epoxides.^[66]^ Even with the low catalyst loadings, this Lewis pair catalytic system comprising TEB and triethylamine (TEA) exhibited significant catalytic activity. A high productivity of as much as 1.2 kg of polymer per g of catalyst under the PO/PA/TEB/TEA stoichiometric ratio of 16,000/2000/4/1 was achieved at 65°C. The Wang group developed a commercial Lewis pairs catalyst system consisting of TEB and organic bases bridging the ROAC of PO/CO_2_ and the ROAC of PO/PA, which afforded poly(propylene phthalate)‐*b*‐PPC with linear or hyperbranched topologies by different CTAs.^[67]^

### 3.3 CO_2_/epoxide/cyclic ester

#### 3.3.1 CO_2_/epoxide/lactone

**SCHEME 11:**
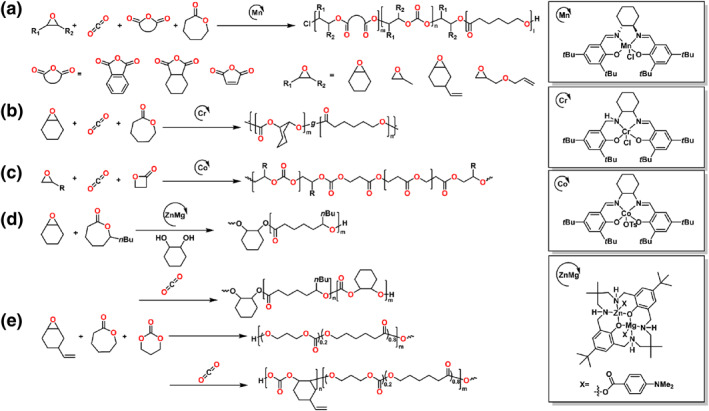
Ring‐opening copolymerization of (a, b, e) CO_2_, epoxide, *ε*‐caprolactone (c) CO_2_, epoxide, *β*‐propiolactone (d) CO_2_, epoxide, *ε*‐decalactone by organometallic compounds.

The Chen group first developed and applied a commercial salen Mn catalyst to synthesize a range of AB‐type, ABA‐type and novel ABC‐type multiblock copolymers through ROCOP of anhydrides, epoxides, CO_2_ and *ɛ*‐CL mixtures (Scheme 11a).^[68]^ The ROCOP of epoxides and CO_2_ could connect the ROAC of anhydrides and epoxides and ROP of lactones as a bridge; this finding demonstrated that the first block enabled the sequential addition of carbonate linkages to the copolymers, which functioned as a macromolecular initiator. The sequence‐controlled ABC block copolymers had *M*_n_s up to 44.6 kg mol^−1^ with *Đ* about 1.23–1.33.

In 2022, Zhou et al. detailed the formation of gradient polycarbonate‐polyester copolymers through an asymmetric Cr complex from monomer mixtures (Scheme 11b).^[69]^ Through tuning reaction conditions or composition ratios, a range of configurations of gradient copolymers could be tuned with their physicochemical properties. The chain structures of the gradient PCHC‐PCL copolymer were confirmed via NMR. Recently, this group reported a new multifunctional degradable material via *ε*‐CL, CO_2_ and CHO.^[70]^ The carbonate units were distributed in a random fashion on the polymer chain due to the ROAC of CHO and CO_2_ and ROP of *ε*‐CL could proceed at the same time. By adjusting the reaction conditions, the obtained polymer had 4 mol% to 33 mol% carbonate content. Therefore, the polymers exhibited a wide series of tunability, such as tough plastics, elastomers, and even adhesives.

The Lu group also reported the controlled random copolymerization of epoxides, CO_2_ and *β*‐propiolactone by regioselective lactone ring opening via a binary catalyst system composed of a trivalent Co complex and an organic base catalyst (Scheme 11c).^[71]^ CO_2_ played a crucial role in inhibiting inter‐ and/or intramolecular transesterification side reactions. The resulting terpolymers had nearly the same compositions at various time points.

Williams and coworkers utilized an organometallic heterodinuclear Zn/Mg catalyst to yield degradable ABA‐type copolymers among biobased *ε*‐DL, CHO and CO_2_ in a one‐pot procedure (Scheme 11d).^[72]^ Using the same catalyst, this group reported the production of the typical poly(vinyl‐cyclohexene carbonate‐*b*‐*ε*‐DL‐*b*‐vinyl‐cyclohexene carbonate) (PvCHC‐PDL‐PvCHC) triblock copolymer.^[73]^ The resulting polymers with high molar masses and narrow dispersities could be attached to carboxylic acids to coordinate metals for toughening CO_2_‐derived copolymer elastomers. Very recently, they combined the ROP of the epoxide and CO_2_ and the ROP of CL and TMC and to prepare poly(carbonate‐*block*‐ester) binders (Scheme 11e).^[74]^ Due to the polymerizations were well‐controlled, a series of polymers were synthesized with varied *M*_n_s or the polycarbonate weight fractions. Moreover, these polymers exhibited the characteristics of polymer electrolyte binders, which can be used in solid composite cathodes.

#### 3.3.2 CO_2_/epoxide/LA

**SCHEME 12:**
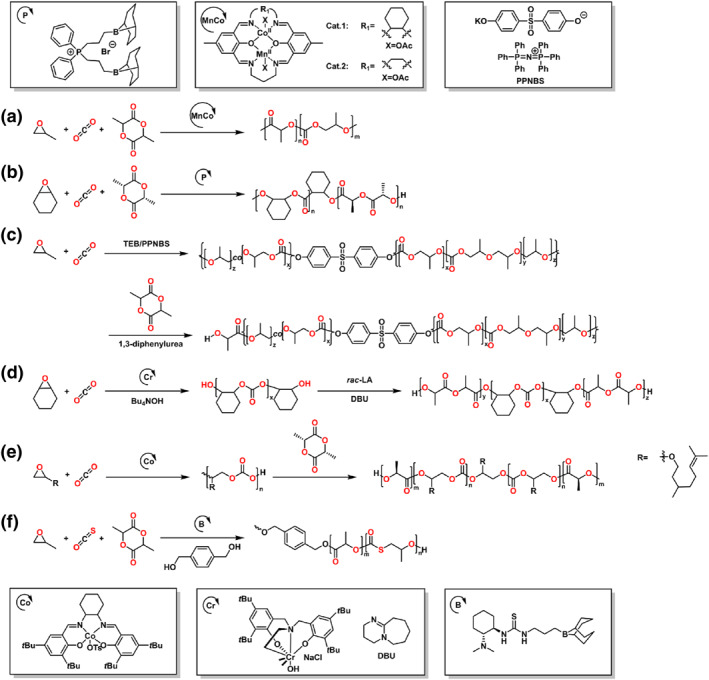
Ring‐opening copolymerization of CO_2_, epoxide and lactide (a, b, f) in one pot (c–e) via two steps.

In 2022, the Chen and Pang group reported the electrochemically controlled switchable copolymerization to obtain a block copolymer consisting of polycarbonate and PLA (Scheme 12a).^[75]^ Depended on the chemical selectivity of different valences of organometallic compounds, the heterometallic Mn/Co complexes demonstrated a composite character of both Salen Mn complex and Salen Co complex. The multi‐block copolymer with different functional groups and microstructures could be further tuned by introducing different monomers. Li and coworkers utilized the CO_2_ as an exogeneous switch reagent through the chemoselective polymerization to synthesize the polycarbonate‐*b*‐polyester multiblock copolymers, which catalyzed through a phosphonium borane Lewis pair catalyst. Tuning the stoichiometric ratio of _L_‐LA and CHO, the obtained diblock copolymer contained the various *M*_n_s (Scheme 12b).^[76]^

Liu et al. described the triblock copolymers composed of PLA and poly(ether carbonate) (Scheme 12c).^[77]^ The poly(ether carbonate) block was obtained by copolymerization of CO_2_ with epoxide through TEB and the PLA blocks could be synthesized by LA and 1,3‐diphenylurea after releasing the CO_2_ in one pot. The molecular weight of the resulting copolymer from the PPPC blocks was 41–48 kg mol^−1^ molar mass maintaining the narrow dispersity. All mechanical parameters, such as toughness and tensile strength, of the triblock copolymers outperformed the commercial polyethylene.

The Kozak group utilized a Cr complex to obtain a polycarbonate diol (Scheme 12d).^[78]^ The polycarbonate diol was used as an initiator to give the ABA‐type copolymer with controlled molar mass and narrow dispersity by the ROP of the *rac*‐LA. Schüttner et al. introduced poly(citronellyl glycidyl ether carbonate) (PCitroGEC), with low glass temperature around −51°C, into poly(ester‐*b*‐carbonate‐*b*‐ester) triblock thermoplastic elastomers (Scheme 12e).^[79]^ The PCitroGEC block was first synthesized by the ROCOP of the CO_2_ with allyl glycidyl ether (CitroGE) using a Co complex. The PLA‐*b*‐PCitroGEC‐*b*‐PLA multiblock copolymers were generated with the DBU‐catalyzed ROP of _L_‐LA.

COS is a similar monomer of CO_2_, consisting of double‐bonded carbon to oxygen and sulfur. The Zhang group reported a three‐site organocatalyst that copolymerized epoxides, *rac*‐LA and COS to obtain a range of sequence‐defined and stereo‐defined block copolymers, which consisted of alternating/regioregular polythiocarbonate and isotactic PLA blocks (Scheme 12f).^[80]^ The obtained multiblock copolymers had a *M*_n_ ∼ 144.8 kg mol^−1^ and *Đ* ∼ 1.1. Regulating the content of polythiocarbonate and PLA blocks could tune the mechanical properties of the obtained block copolymers. Further, these block copolymers showed impressive ductility and toughness due to their high molecular weights and isotactic PLA fragments.

### 3.4 CO_2_/epoxide/vinyl monomer

**SCHEME 13:**
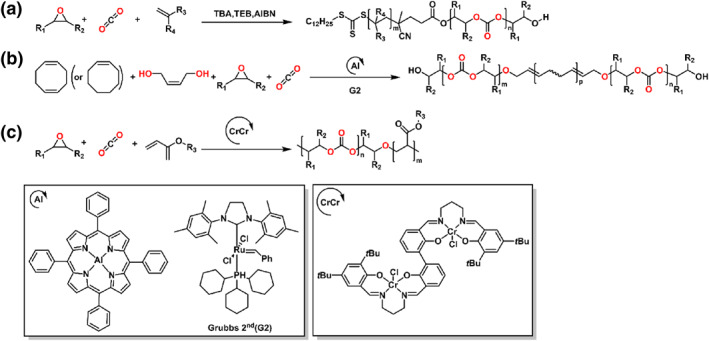
Ring‐opening copolymerization of CO_2_, epoxide and vinyl monomer (a) by organocatalysts and (b, c) by organometallic compounds.

Patil et al. reported a one‐pot method to synthesize multiblock copolymers comprising polyvinyl block with CO_2_‐based block by TEB (Scheme 13a).^[81]^ The polymerization consisted of RAFT of the vinyl monomers and ROAC of epoxides and CO_2_. 4‐Cyano‐4‐[(dodecylsulfanylthiocarbonyl) sulfanyl] pentanoic acid (TTC‐COOH) was used as an initiator in the ROAC of epoxide and CO_2_ and a CTA in the RAFT. All these diblock and triblock copolymers had narrow dispersities and unimodal distributions of molar masses. Qin and coworkers demonstrated the formation of constructing CO_2_‐based block copolymers through an Al complex and a trithiocarbonate/carboxy bifunctional CTA, which combined RAFT and ROCOP in the one‐step.^[9]^ Moreover, because of the thermo‐responsive of the block copolymers, these copolymers had the capability to self‐assemble into micelles. In 2024, this group demonstrated the synthesis of CO_2_‐based polycarbonates comprising ROMP with ROCOP from epoxides, CO_2_ and cycloalkenes through a one‐pot route (Scheme 13b).^[82]^ The polycarbonate‐*b*‐polyalkenamer‐*b*‐polycarbonate exhibited controllable *M*_n_s with narrow dispersities.

Recently, Lu and coworkers utilized dinuclear Cr complexes to directly synthesize the diverse polyacrylate‐based copolymers, covering polyether‐*b*‐polyacrylate, polycarbonate‐*b*‐polyacrylate, polythiocarbonate‐*b*‐polyacrylate, polyester‐*b*‐polyacrylate and polyurethane‐*b*‐polyacrylate (Scheme 13c).^[83]^ The synergistic bimetallic catalysis allowed the alkoxide ion to bind to one Cr‐center nucleophilic attack at the acrylate enchained by the adjacent Cr‐center in the catalytic cleft, thus intriguing the homopolymerization of acrylates. The DOSY NMR confirmed that the obtained polymers were copolymers. Among various organometallic compounds and organocatalysts, the polyolefin‐*b*‐polyether block copolymers could only be directly synthesized from the copolymerization of epoxides and olefins via dinuclear Cr complexes.

### 3.5 CO_2_/epoxide/other monomers

#### 3.5.1 CO_2_/epoxide/OPA

**SCHEME 14:**
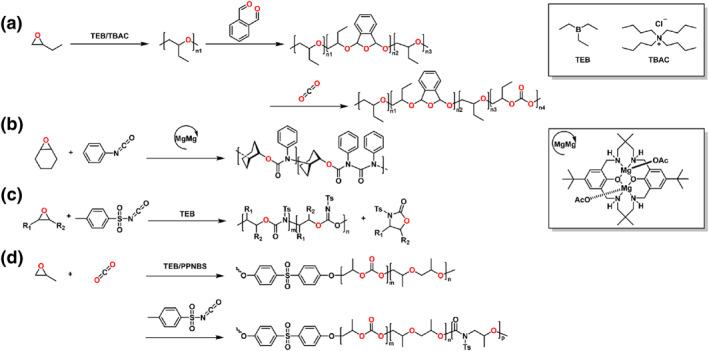
Ring‐opening copolymerization of (a) CO_2_, epoxide and *O*‐phthalaldehyde and (b–d) CO_2_, epoxide and isocyanate.

*O*‐phthalaldehyde (OPA), a new class of monomers, could be polymerized through either cationic or anionic approaches. Because of the high strength and thermal stability of polyacetal, it is an interesting segment to introduce into block copolymers. In 2022, the Feng and Gnanou group demonstrated that the anionic copolymerization of OPA with epoxides could be carried out at room temperature by TEB.^[84]^ Recently, the Chen and Pang group reported that multiblock copolymers from epoxides, OPA and CO_2_ were synthesized by using a Lewis pair of tetrabutylammonium chloride and TEB (Scheme 14a).^[85]^ The ABC‐type triblock, ABAC‐type tetrablock and ABCBC‐type pentablock block copolymers were prepared by composing of polycarbonate, polyether and polyacetal. Moreover, the obtained copolymers had a high thermal degradation temperature.

#### 3.5.2 CO_2_/epoxide/isocyanate

In 2020, the Adriaenssens group successfully achieved the ROCOP of epoxides and isocyanates (Scheme 14b).^[86]^ The resulting multiblock copolymers for AB‐ and ABB‐type constituted polyurethanes and the polyallophanates had an unprecedented backbone structure. Subsequently, the Feng and Gnanou group reported a direct method for the ROAC of epoxides and *p*‐tosyl isocyanate (TSI), utilizing TEB as a catalyst and onium salts as initiator, the catalysis system was also useful for the ROCOP of the CO_2_/epoxides (Scheme 14c).^[87]^ In 2023, their group synthesized triblock copolymers from CO_2_, PO and TSI through a difunctional initiator and TEB (Scheme 14d).^[88]^ The ABA triblock copolymers consisted of two poly(*N*‐tosyl propylene urethane) (PTPU) blocks with a PPPC middle block. The triblock copolymer showed higher toughness than commercial polyolefins, which had 80–85 wt % carbonate in the PPPC block and 28 wt % hard PTPU blocks.

## 4 RO(CO)Ps INVOLVING OTHER MONOMERS

### 4.1 *O*‐carboxyanhydride

*O*‐carboxyanhydrides (OCAs) with functional groups were used to the synthesis of functionalized polyesters in recently years, which were prepared from amino or hydroxy acids from renewable resources. Poly(*α*‐hydroxy acids) and PLA exhibited inferior mechanical and thermal properties compared to commodity polyolefins because their polyester backbones lacked side‐chain functional groups.^[89]^ Therefore, the OCAs were introduced into the ROCOP of other monomers to obtain the functional multi‐block copolymers.

#### 4.1.1 Epoxide/OCA

**SCHEME 15:**
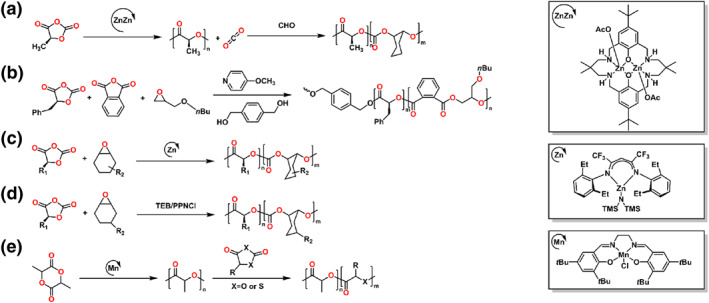
Ring‐opening copolymerization of (a) CO_2_, epoxide, *O*‐carboxyanhydride; (b) epoxide, anhydride, *O*‐carboxyanhydride; (c, d) epoxide, *O*‐carboxyanhydride and (e) lactide, *O*‐carboxyanhydride.

The Williams group first demonstrated the formation of functionalized poly(ester‐*b*‐carbonates) through selective polymerization in the mixtures of CHO and _L_‐lactide‐OCA, utilizing a Zn complex in a one‐pot procedure (Scheme 15a).^[90]^ The Li group showed the selective copolymerization among epoxides, anhydrides and OCAs in one‐pot by an organocatalyst in 2021 (Scheme 15b).^[91]^ The ABCBA multiblock copolymer, which composed of three blocks, was obtained via a combination of ROP of LA, ROCOP of PA/epoxides and the ROP of OCA without external monomer addition in one‐pot.

In 2022, the Tong group used a Zn complex at room temperature to synthesize poly(ester‐*b*‐carbonates) by the copolymerization of epoxides with OCAs (Scheme 15c).^[92]^ The block copolymers had high *M*_n_s up to 200 kg mol^−1^ with narrow dispersities. Moreover, the copolymers without tapered structure outperformed some commodity polyolefins,which exhibited excellent ductility (fracture strain >400%) and high tensile stress (fracture stress >20 MPa). At the same time, the Wang and Tao group reported the formation of poly(ester‐*b*‐carbonates) through onium salt/TEB Lewis‐pair organocatalyst from a mixture of OCAs and epoxides (Scheme 15d).^[93]^ The ABCB‐type tetrablock copolymers were prepared using two steps. Compared with the Zn complex, the Lewis‐pair organocatalyst for the tandem copolymerization gave polymers with low *M*_n_s.

#### 4.1.2 Cyclic ester/OCA

The Chen and Pang group exhibited the formation of multiblock polyesters via chemoselective ROCOP of OCAs with LA (Scheme 15e).^[7]^ The controlled self‐switchable polymerization was depended on the insertion of CO_2_ generated from the ROP of OCAs by a Mn complex. Varying OCAs with different pendant groups were utilized to synthesize a range of multi‐block copolymers. Moreover, the antibacterial PLA could be synthesized through modifying copolymers that contained propargyl groups with quaternary ammonium groups. In 2022, this group used the same strategy to explore the sulfur‐containing carboxyanhydride to polymerize multiblock thiopolyesters. A five‐block polyester/polythioester copolymer was obtained with *M*_n_ = 12.4 kg mol^−1^ and *Đ* = 1.20.^[94]^

### 4.2 Cyclic ester

#### 4.2.1 *δ*‐Caprolactone

In 2022, Li and coworkers demonstrated the controlled ROP of commercially available *δ*‐caprolactone (*δ*‐CL) through binary catalysts consisting of urea and strong base (Scheme 8).^[95]^ The well‐defined PLLA‐*b*‐P(*δ*‐CL)‐*b*‐PLLA copolymers were synthesized through the ROP of _L_‐LA and *δ*‐CL. These triblock copolymers behaved as thermoplastic elastomers, which exhibited the excellent ultimate elongation, elastic recovery and tensile strength. Moreover, these resulting copolymers could recover with high yields through the chemical recycling. Subsequently, they further used a series of alkyl‐*δ*‐lactones with varying alkyl substituent lengths to copolymerize with _L_‐LA to obtain well‐controlled multiblock copolymers. Moreover, these copolymers could be utilized as pressure‐sensitive adhesives after polymerization without the other steps.^[96]^

**SCHEME 16:**
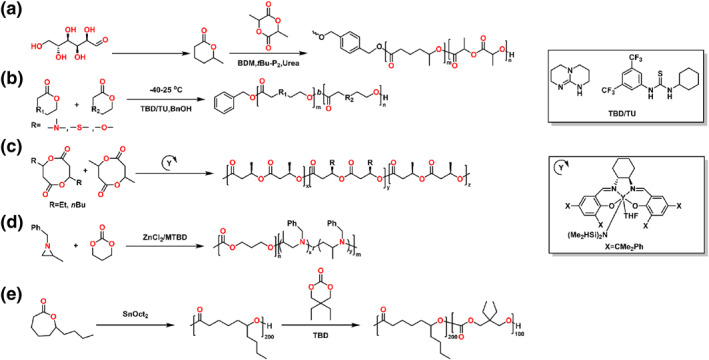
RO(CO)P of (a–c) cyclic ester and (d, e) trimethylene carbonate.

#### 4.2.2 *δ*‐Valerolactone

The Zhang and Wang group designed a range of *δ*‐valerolactone (*δ*‐VL) based monomers, where the C‐5 was substituted by N, S and O (Scheme 16b).^[97]^ The three VL‐based monomers showed different homopolymerization behaviors in the study of polymerization kinetics and thermodynamics. Therefore, the various sequence‐controlled block, random, gradient structures copolymers could be achieved through the ROCOP of two of the three *δ*‐VL based monomers enabled at different temperatures. Moreover, the random or gradient copolymer could be completely depolymerized to their original monomers.

#### 4.2.3 8‐Membered diolide

The Chen group synthesized 8‐membered dialkyl diolides to obtain a variety of stereoregular aliphatic polyhydroxyalkanoates (PHAs) to overcome the limitations of the biosynthesis of PHAs (Scheme 16c).[^98^, ^99^] In 2023, they reported a stereoselective polymerization route to synthesize the triblock copolymers PHAs by racemic Y complexes.^[100]^ The resulting copolymers had high *M*_n_s as much as 238 kg mol^−1^ and narrow dispersities. Due to the varying degrees of crystallinity, the obtained triblock copolymers exhibited adjustable mechanical properties, exhibiting characteristics of either a semicrystalline thermoplastic elastomer or a tough and strong thermoplastic elastomer.

### 4.3 Trimethylene carbonate

#### 4.3.1 TMC/aziridine

Guo et al. comprised polycarbonate and acidified polyamine segments through the ROP of TMC and aziridine (Scheme 16d).^[101]^ The chain propagation was achieved simultaneously through ROP of cyclic carbonates and the cationic and anionic ROP of aziridine. The resulting block copolymers afforded amphiphilic polymers, which could self‐assemble to form polysomes after acidification.

#### 4.3.2 TMC/cyclic ester

The Odelius group demonstrated the formation of three different block copolymers by combining LA, *ε*‐DL, 2,2‐diethyltrimethylene carbonate (Et_2_TMC) and TMC (Scheme 16e).^[102]^ Based on different cyclic monomer structures and solvent properties, the recyclable ABA block copolymers were designed with varying material properties. Further, the generated copolymers could be recycled selectively to the corresponding monomers.

## 5 CONCLUSION

A series of the sequence controlled multiblock polyesters and polycarbonates have been obtained by the heterocycle ROP or ROCOP, depending on the development of the effective catalysts and the new heterocycle monomers. Various heterocycle monomers and monomer combinations provide the diverse structure and functionality of the polyesters and polycarbonates. To date, most of the block polyesters and polycarbonates have been generated through the addition of monomers sequentially in the polymerization. In recent years, the switchable polymerization in block polyesters and polycarbonates synthesis have attracted considerable attention because this approach enables to connect the different catalyst cycles in a mixture of monomers in one‐pot. Otherwise, although the organometallic compounds still play a dominant role in the synthesis of block polyesters and polycarbonates, they often need tedious synthesis steps and expensive costs. Organocatalysts have been widely utilized in the ROP and ROCOP, and they have the potential to synthesize block polyesters and polycarbonates.

Moreover, there are several opportunities for further research and discovery in the formation of the well‐defined block polyesters and polycarbonates. Firstly, the synthesis of high molecular weight (*M*_n_ > 100 kg mol^−1^) block polyesters and polycarbonates remains challenging. Undesired transesterification side reactions at high conversion, hydrolyzed anhydride or adventitious water in the polymerization system lead to the low molar mass of the resulting block polyesters and polycarbonates.^[1]^ Otherwise, current catalyst/cocatalyst catalytic systems used for the formation of block polyesters and polycarbonates generally show poor activities at low catalyst loadings, which makes it hard to synthesize the high‐molecular‐weight block copolymers.[^13^, ^103^] Secondly, stereoregular and stereosequence‐controlled polymers show enhanced physical and mechanical properties relative to their atactic counterparts.^[104]^ Therefore, incorporating the stereochemistry into block polyesters and polycarbonates will improve the properties of the obtained polymer. Third, the polyesters and polycarbonates have the potential ability of chemical sustainability, thus the block copolymers based on polyesters and polycarbonates are supposed to recycle or upcycle. Finally, the sequence‐controlled block polyesters and polycarbonates should be used for smarter applications in different fields.

In conclusion, further investigation is needed in the sequence‐controlled block polyesters and polycarbonates derived from controlled heterocycle ROP or ROCOP. Further research is suggested to generate the diverse structure and functionality of the block polyesters and polycarbonates to form smart molecules.

## CONFLICT OF INTEREST STATEMENT

The authors declare no conflicts of interest.
